# Glycaemic control in ICUs in large English hospitals: a follow-up telephone survey

**DOI:** 10.1186/cc10784

**Published:** 2012-03-20

**Authors:** CR Bullock, A Pang, A Routledge, I Mackenzie

**Affiliations:** 1 Queen Elizabeth Hospital Birmingham, UK

## Introduction

Following van den Berghe's landmark paper in 2001 (Leuven study) [1], the critical care community became very interested in 'tight' glycaemic control [2]; however, recent negative studies have dampened this interest [3]. In view of more recent analyses, which offer possible explanations for equivocal results [4], it is possible there will be renewed interest in glycaemic control. The purpose of this survey is to assess the utilisation of tight glycaemic control protocols in a sample of ICUs in England, as a reflection of current UK intensive care practice.

## Methods

We identified 171 large acute hospital trusts, of which 87 were randomly selected. Of these, 85 had ICUs, which were contacted by telephone. The senior nurse in charge at the time was asked whether their ICU used a protocol for the management of blood glucose, and what were the upper and lower target limits.

## Results

A blood glucose protocol was used in 87.1% of ICUs surveyed. Of these, the median lower limit of allowed blood glucose concentration was 4.0 mmol/l (range 3.0 to 7.0), with an upper limit of 8.0 mmol/l (range 6.0 to 12.0). Only 22 ICUs (25.9%) had a target range similar to the Leuven study. A further 34 ICUs used a lower limit similar to the Leuven study, of 4.0 to 4.5 mmol/l, but had a higher upper limit. This is reflective of the general opinion from the nurses contacted, that a tight protocol is difficult to achieve, can result in hypoglycaemia, and has been recently relaxed in many departments.

## Conclusion

Our data suggest that glycaemic control has, to a very large extent, been accepted as a standard of care in the UK, although in most ICUs this does not constitute tight glycaemic control. The full benefit of tight glycaemic control, achieved by minimisation of mean glucose, glucose variability and episodes of hypoglycaemia, will not be achieved until robust techniques for continuous, or semi-continuous, blood glucose measurement are available.
